# Supplementary material for: Atomistic mechanism of coupling between cytosolic sensor domain and selectivity filter in TREK K2P channels
Source: Nat Commun. 2024 May 31;15:4628. doi: 10.1038/s41467-024-48823-y (PMC11143257; doi:10.1038/s41467-024-48823-y)
Supplement: Supplementary file 1 — Supplementary Information [file 41467_2024_48823_MOESM1_ESM.pdf]

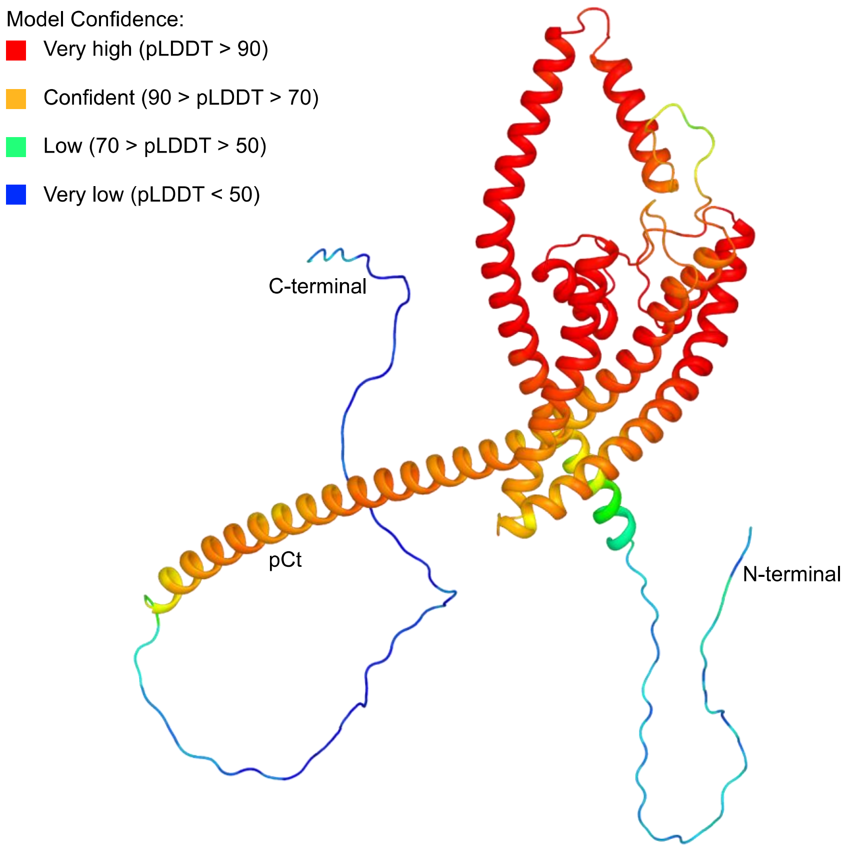

Supplementary Fig. 1. Predicted structure of KCNK2 (TREK-1) channel using AlphaFold2[1] showing the conformation of structurally undetermined region of the C-terminus.

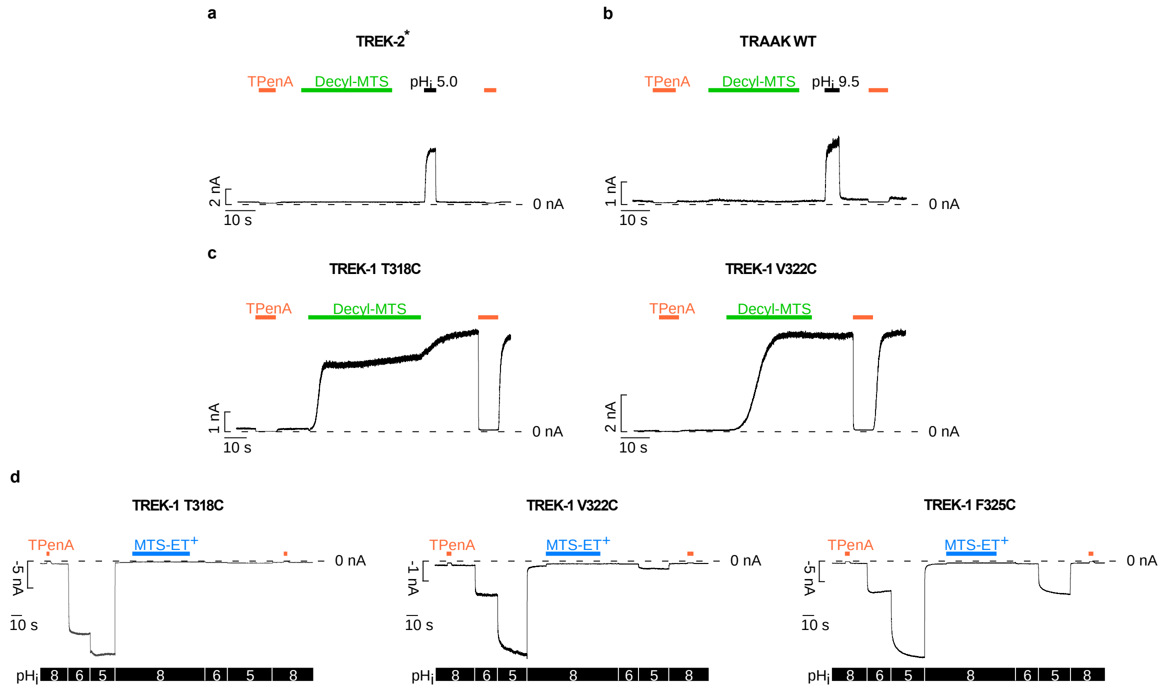

Supplementary Fig. 2. Validation of membrane tethering assay with cysteine modifying probes. (a,b) Example recordings of TREK-2* (a) and WT TRAAK channels (b) in a symmetrical K^+ gradient at +40 mV and pH 7.4 showing robust activation with intracellular pH 5.0 or 9.5, respectively but no effect on the application of 100 µM decyl-MTS. **(c)** Same recordings as in (a,b) for T318C and V322C mutant TREK-1 channels showing strong and irreversible activation with 100 µM decyl-MTS. **(d)** Example recordings in a symmetrical K^+ gradient at -80 mV for T318C, V322C and F325C mutant TREK-1 channels showing robust activation upon intracellular acidification and channel inhibition with 1 mM MTS-ET^+.

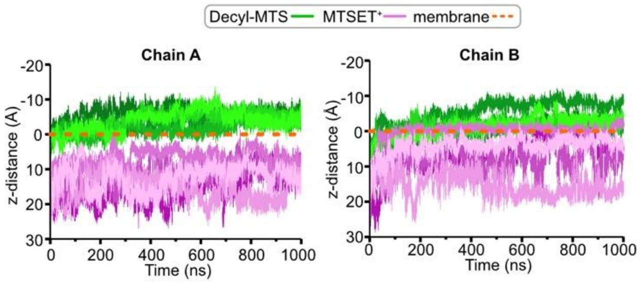

Supplementary Fig. 3. Distance between MTS ligand and the membrane derived from the simulations of MTS-modified TREK-2* simulations under -200 mV transmembrane voltages. The distance (Dz component of the vector) between a chosen atom of the ligand (C12 for decyl-MTS, N2 for MTS-ET$^+$) and the membrane plotted against simulation time. The membrane was defined as a plane by selecting the center of geometry of phosphorus atoms located in the cytosolic part of the POPC lipid bilayer.

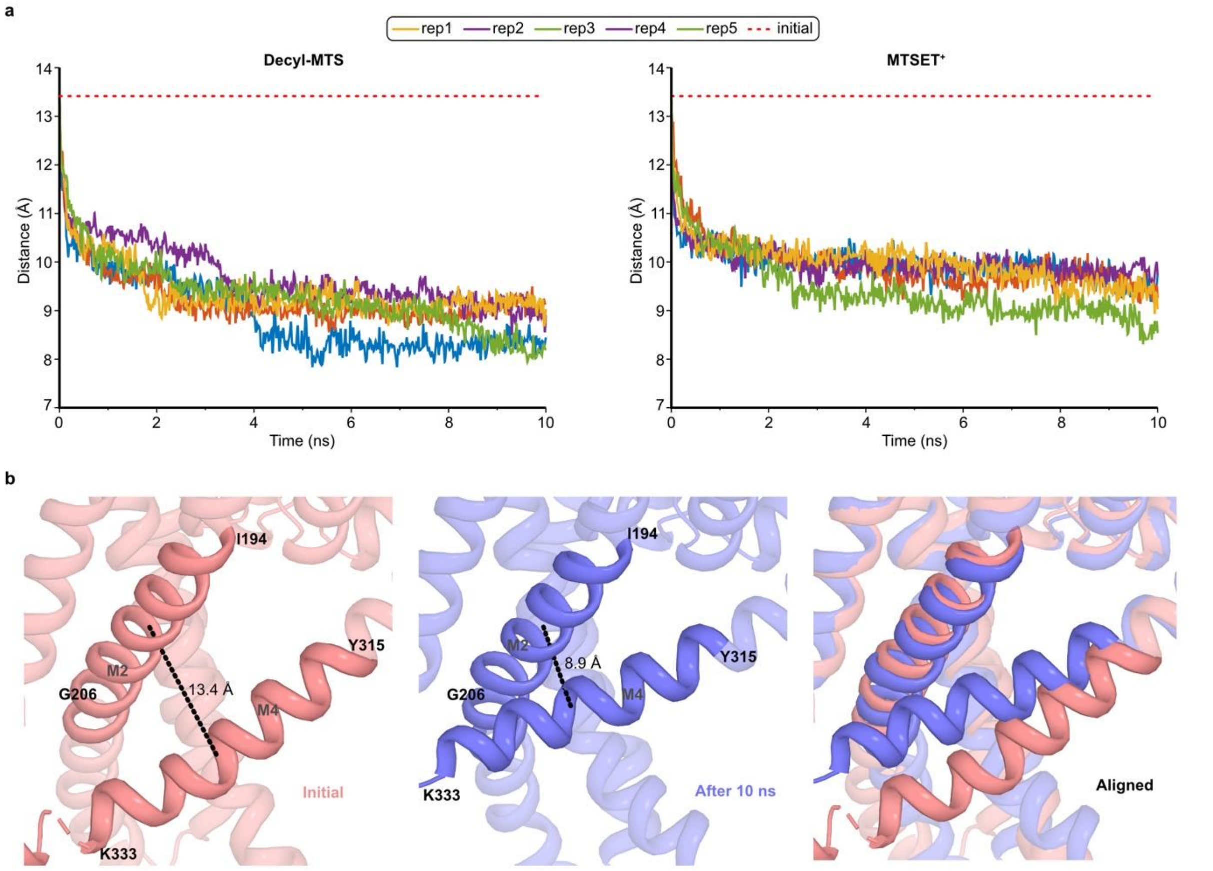

Supplementary Fig. 4. Interchain M2-M4 distances derived from ABMD simulations. (a) Distance between center of mass of M2 (carbon alpha atoms from I194 to G206) and M4 (carbon alpha atoms from Y315 to K333) for decyl-MTS and MTS-ET$^+$. Red dashed line shows the distance in the initial down-state structure between M2 and M4. **(b)** Selected initial, final and overlaid snapshots and the distance between M2 and M4. 5 runs of 10 ns ABMD simulations were performed for for decyl-MTS and MTS-ET$^+$, respectively.

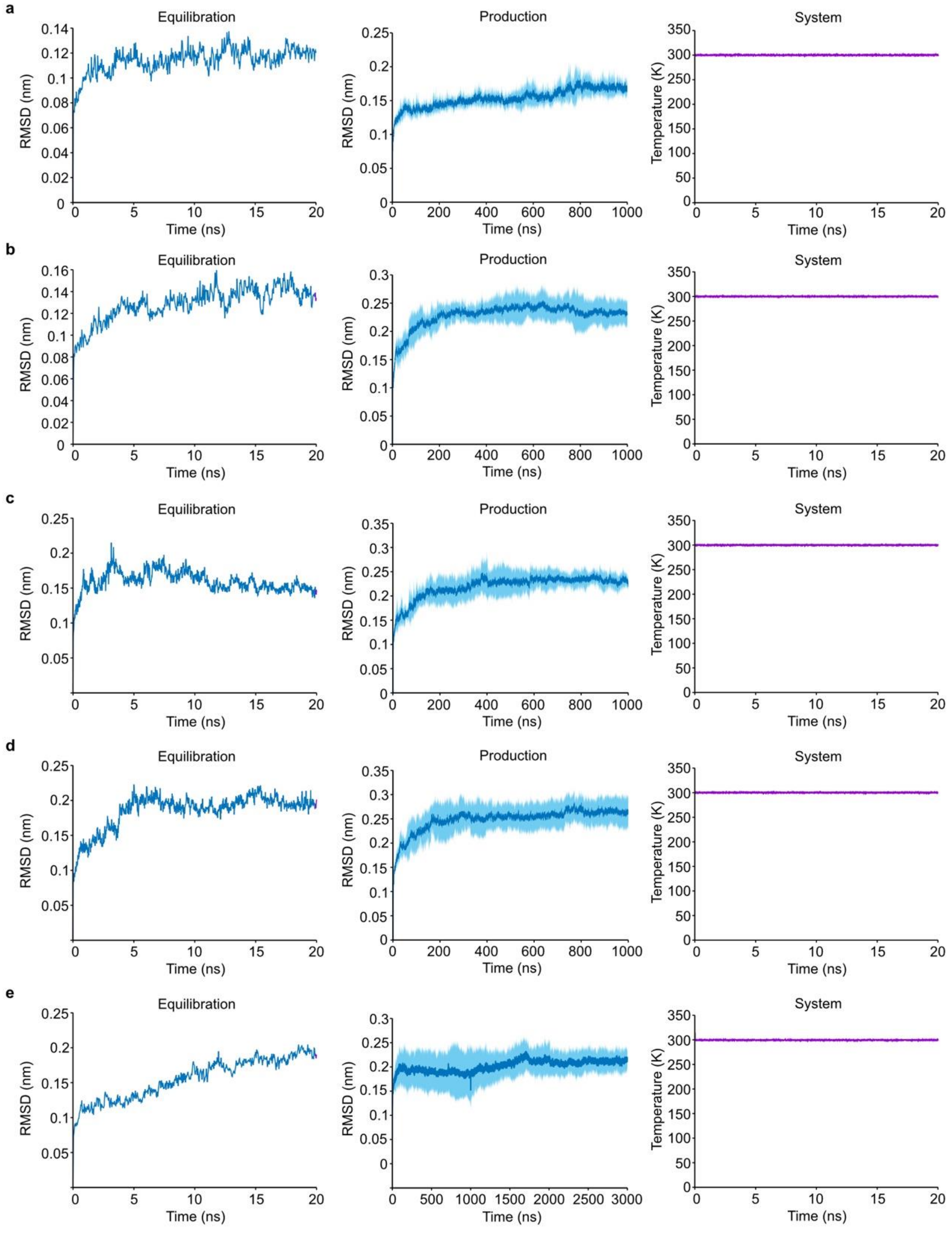

Supplementary Fig. 5. RMSD analysis of the MD data. The time course of root-mean-square deviation (RMSD) during the 20 ns equilibration (first colomn), the averaged RMSD during production simulations (second coloum), and the temperature of the system during equilibration for **(a)** apo up-state, **(b)** apo down-state, **(c)** decyl-MTS-attached down-state, **(d)** MTS-ET^{+}-attached down-state, and **(e)** phosphorylated down-state systems.

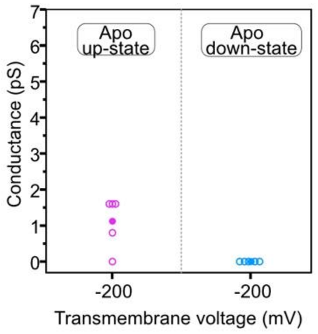

Supplementary Fig. 6. Conductance from TREK-2* simulations. Ion conductance derived from apo TREK-2* simulations in its up- (PDB ID: 4BW5)[2] and down-state (PDB ID: 4XDJ)[2] at -200 mV transmembrane potential. Filled and empty circles represent mean and individual ion conductance, respectively.

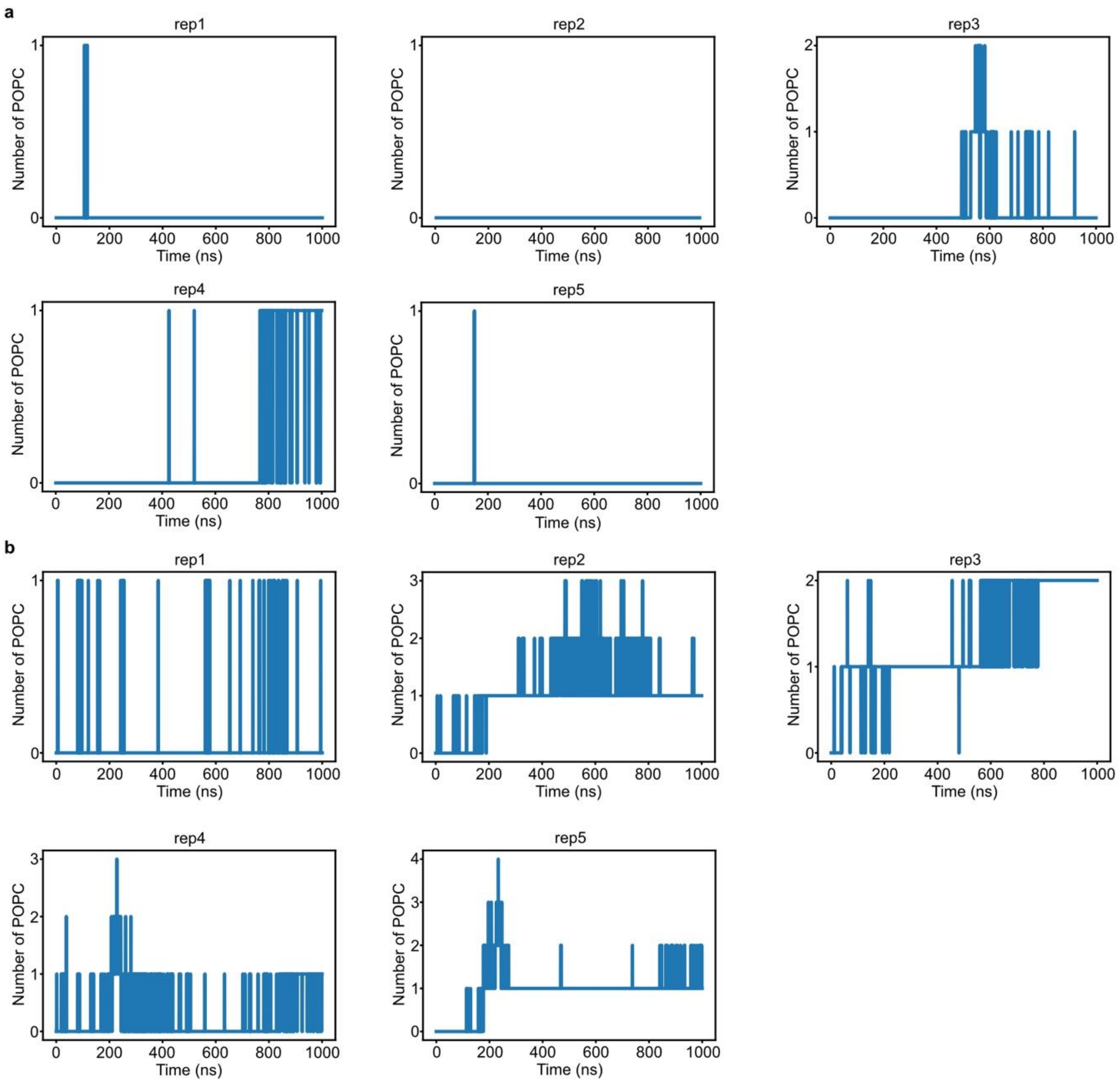

Supplementary Fig. 7. Number of lipids occupying the pore during up- and down-state simulations. Number of POPC lipids occupying the pore (defined in the analysis as a cylinder) for **(a)** up- and **(b)** down-state TREK-2 simulations over the course of the simulation time. In the rep2 of up-state simulations, no lipid entered the pore. The center of the cylinder is positioned 10 Å below the center of mass of the selectivity filter residues T171 and T280, located near the S4 ion-binding site. The height of the cylinder is defined 40 Å and radius is defined 8 Å.

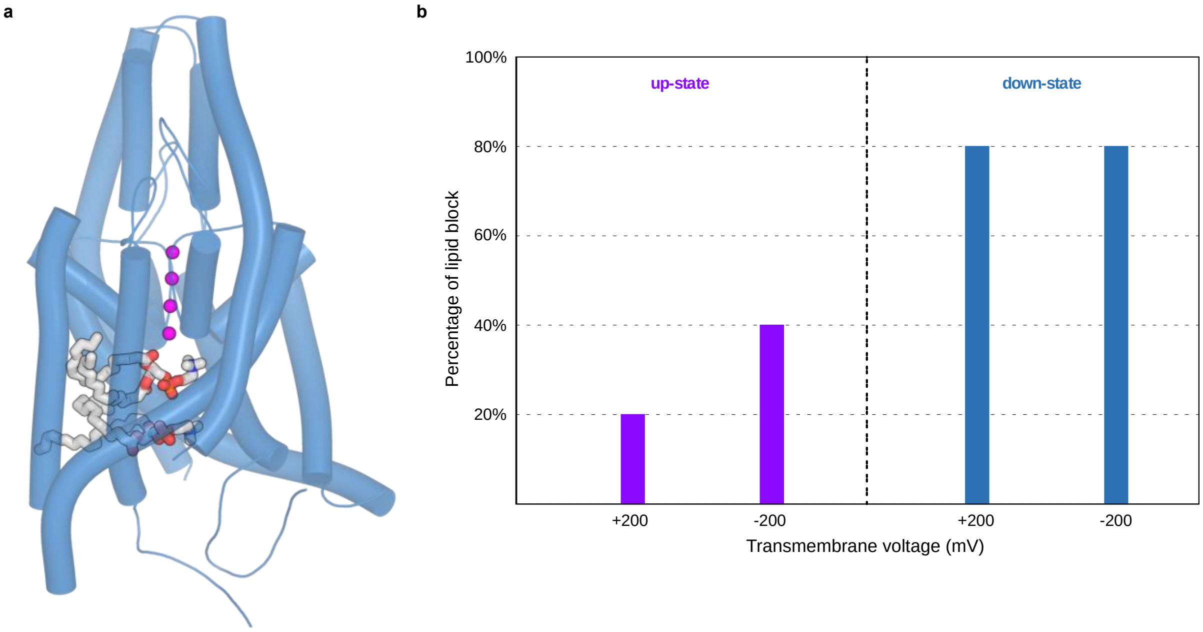

Supplementary Fig. 8. Lipid blockage of the inner pore. **(a)** Selected end-snapshot from down-state simulations showing SF with K^+ ions (magenta) and the lipids (gray) blocking the fenestration side and the pore region. **(b)** Percentage of the simulations showing a lipid occupation in the inner pore of the TREK-2* channel during simulations. End-snapshots of each replica were used to analyze the number of blockage events.

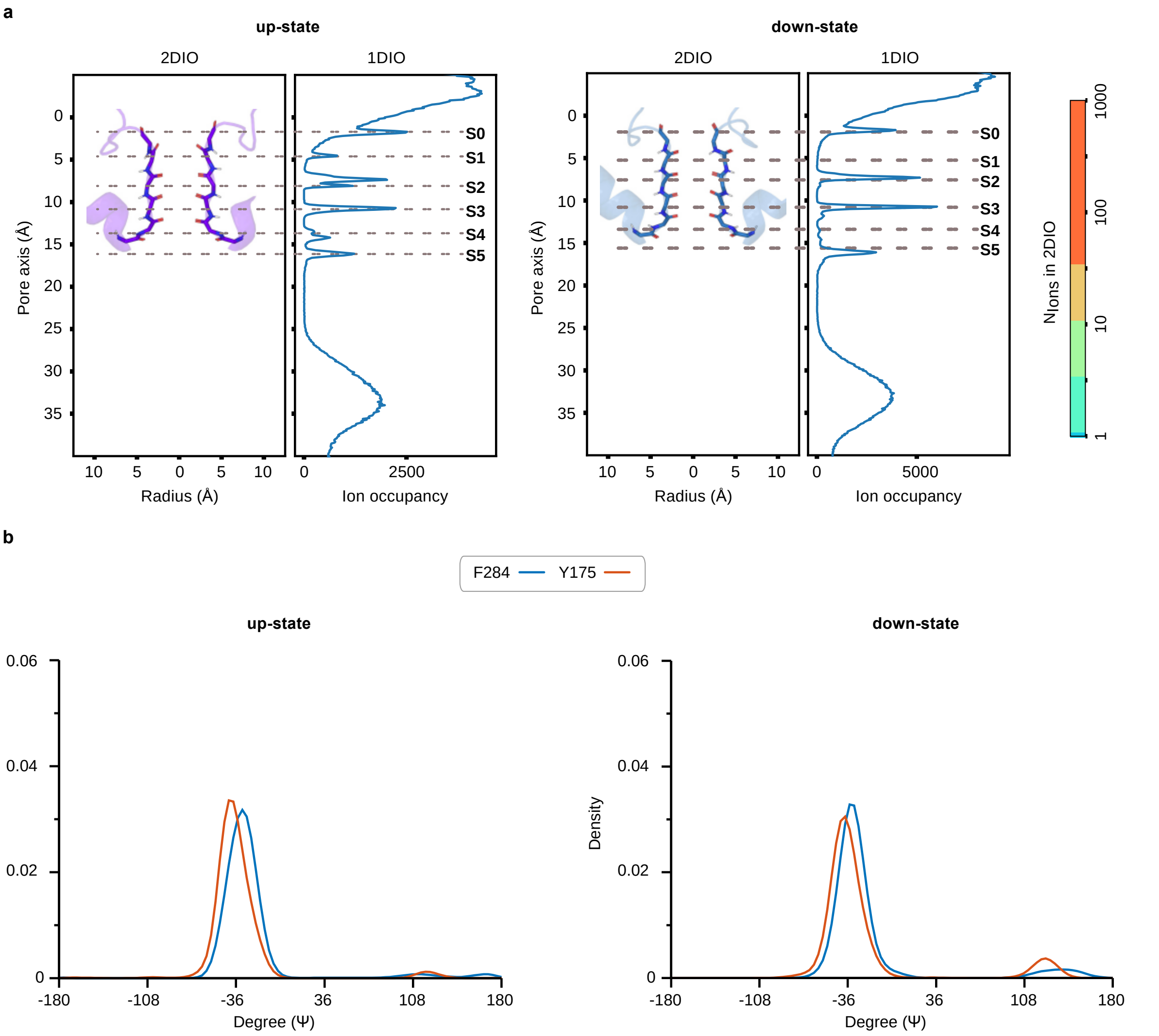

Supplementary Fig. 9. One- and two-dimensional ion occupancies. (a) One- and two-dimensional ion occupancy within the SF for combined up-state (left) and non-conductive down-state (right) TREK-2* simulations at -200 mV. The radial area of the pore was defined and ions passing along the pore axis (D_z) were calculated from the simulations. Based on the volume change along the radius, the occupancy of ions was normalized per 0.001 $Å^3$ per 1 μs. The center of mass of the SF backbone atoms was located on the 7 Å point along the pore axis. Ion binding sites along the pore axis were indicated with dashed lines. **(b)** Psi angle (ψ) distributions of F284 and Y175 residues from the combined up-state (left) and down-state (right) simulations at -200 mV transmembrane potential.

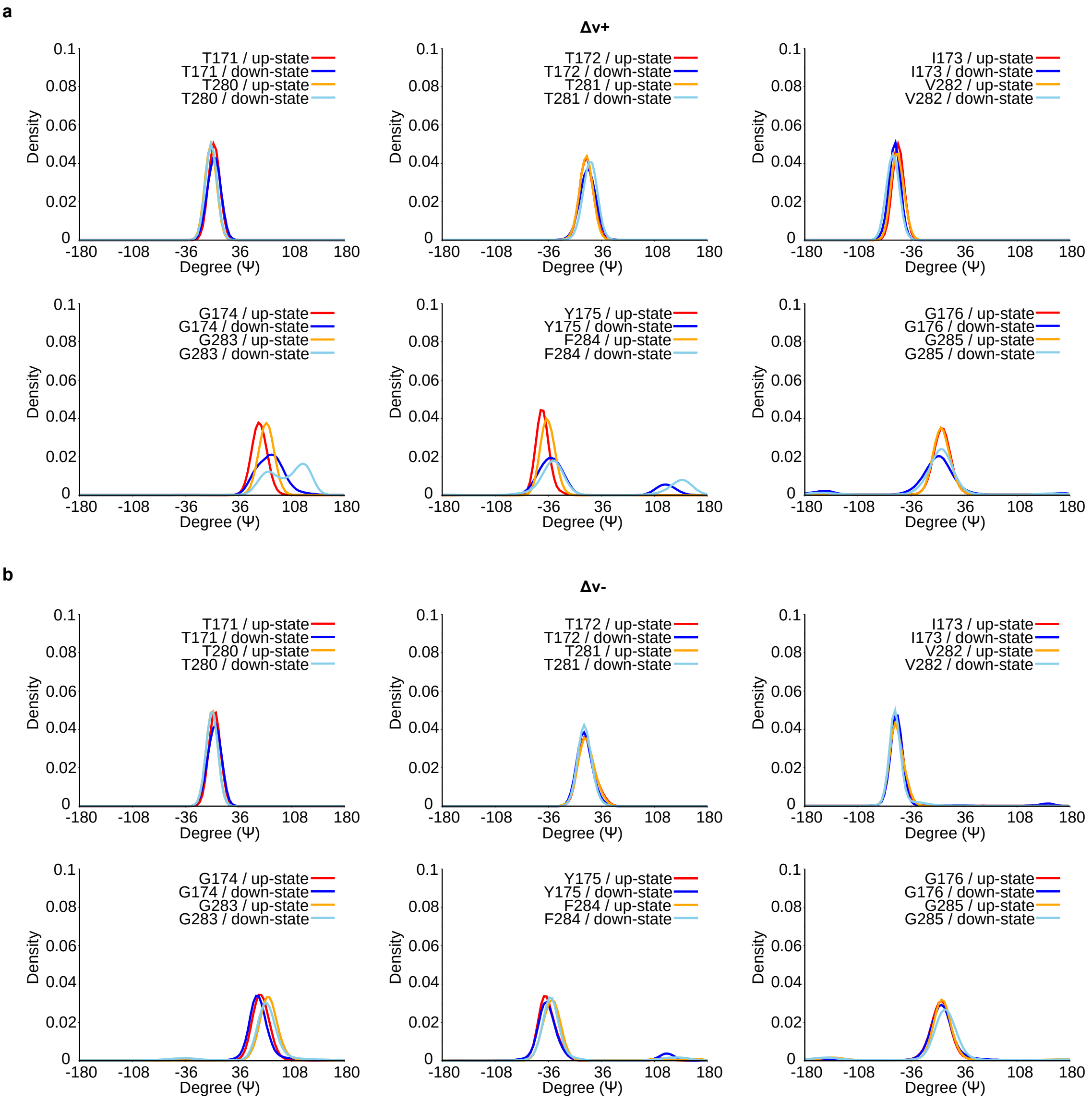

Supplementary Fig. 10. Conformational analysis of the SF residues. Psi angle (ψ) distributions of SF residues from the 5 runs of 1 μs simulations of up-state and down-state TREK-2* at **(a)** +200 mV and **(b)** -200 mV transmembrane potential.

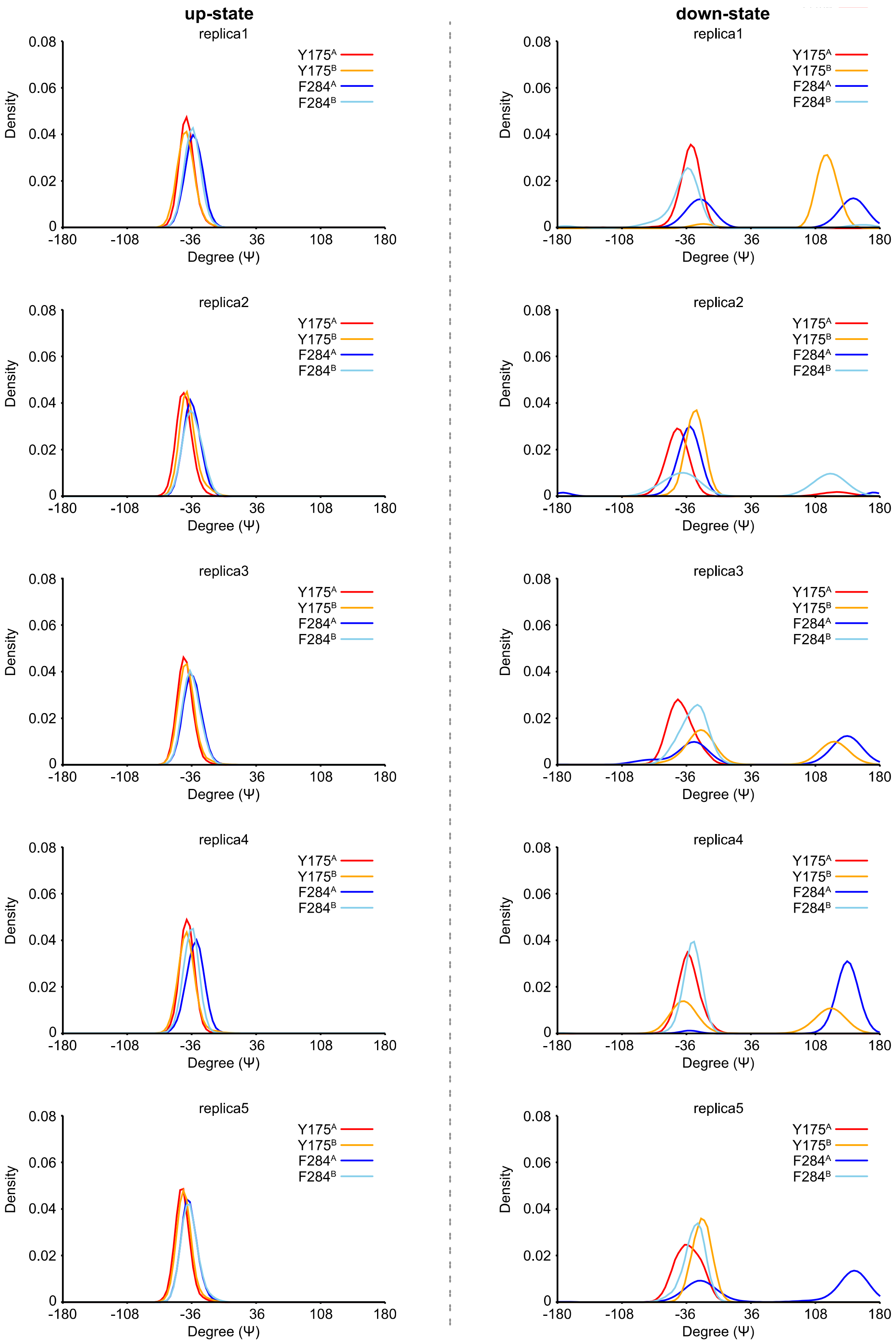

Supplementary Fig. 11. Conformational analysis of residues near S1 ion binding site. Psi angles (ψ) of F284 and Y175 in chain A and chain B over the time from the up-state TREK-2* simulations at +200 mV transmembrane potential (left) and non-conductive down-state TREK-2* simulations at +200 mV transmembrane potential (right). Individual simulation runs were carried out with AMBER99sb[3] for 1 μs.

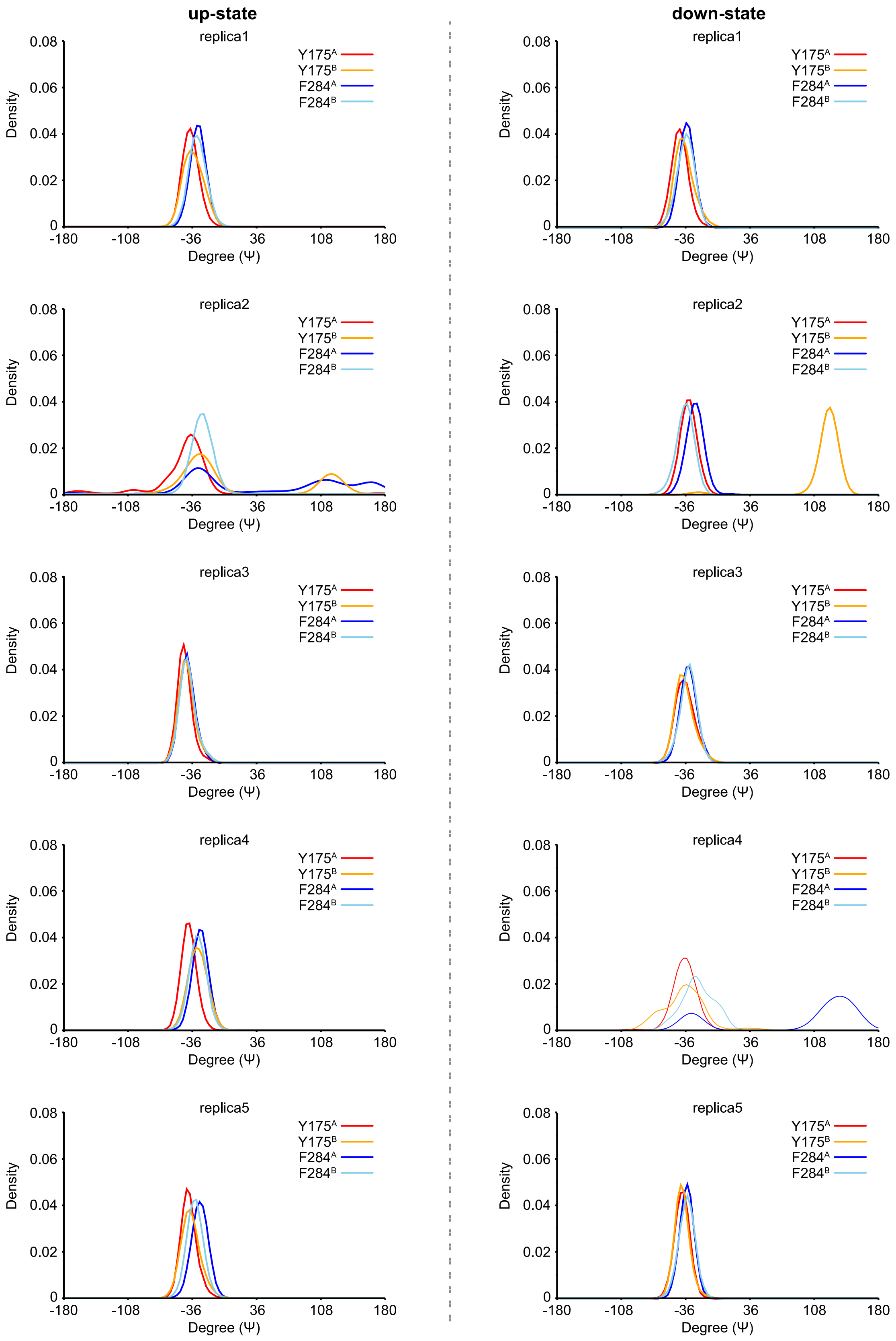

Supplementary Fig. 12. Conformational analysis of residues near S1 ion binding site. Psi angles (ψ) of F284 and Y175 in chain A and chain B over the time from the up-state TREK-2* simulations at -200 mV transmembrane potential (left) and non-conductive down-state TREK-2* simulations at -200 mV transmembrane potential (right). Individual simulation runs were carried out with AMBER99sb[3] for 1 µs.

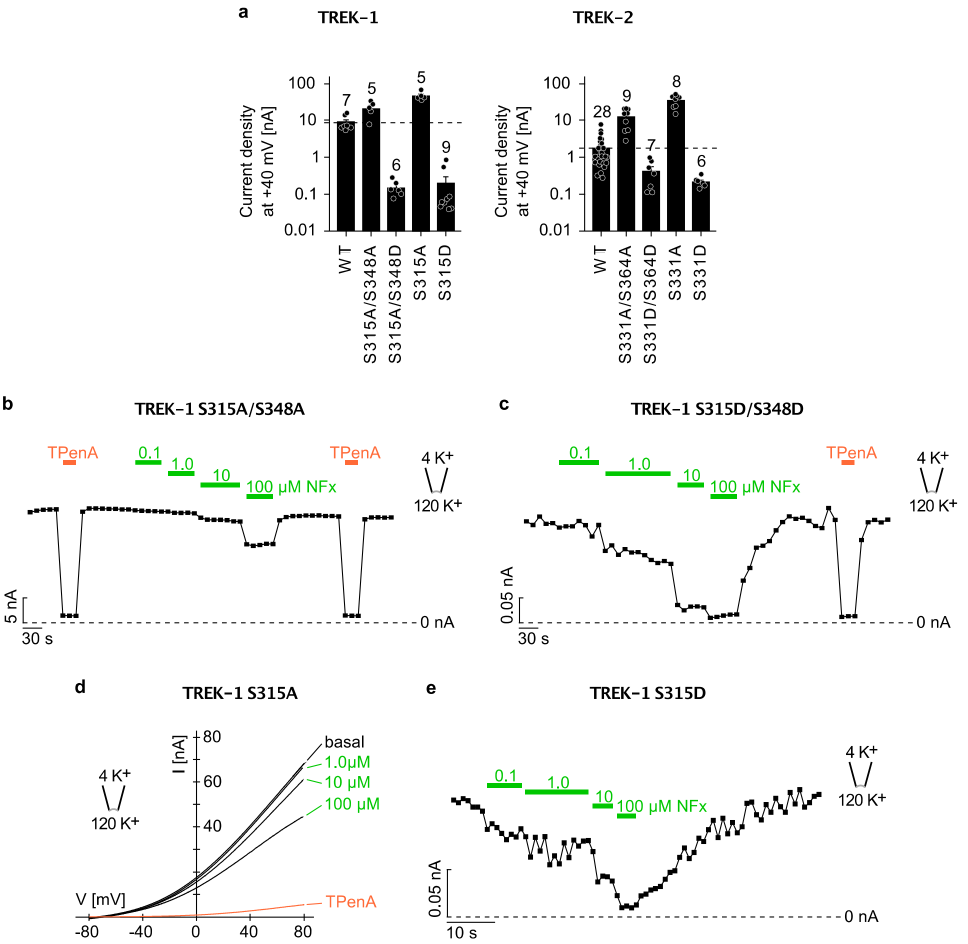

Supplementary Fig. 13. NFx sensitivity of TREK-1 (de-)phosphorylation mimicking mutant channels. (a) Current densities of WT and mutant TREK-1/-2 channels illustrating the gain-of-function or loss-of-function effect of the (de-)phosphorylation mimicking mutations. **(b,c)** Analysis at +40 mV from measurements as in (d) for S315A/S348A (b) or S315D/S348D double mutant TREK-1 channels (c) showing an altered apparent affinity of NFx in contrast to WT. **(d)** Example recording of dephosphorylation at PKC site mimicking S315A mutant TREK-1 channels in an asymmetrical K^+ showing a decreased dose-dependent inhibition with NFx with indicated concentrations (green traces) and an almost full block with 1 mM TPenA (orange trace). (**e**) Analysis at +40 mV from measurements as in (d) for S315D mutant TREK-1 channels showing an altered apparent affinity of NFx in contrast to WT.

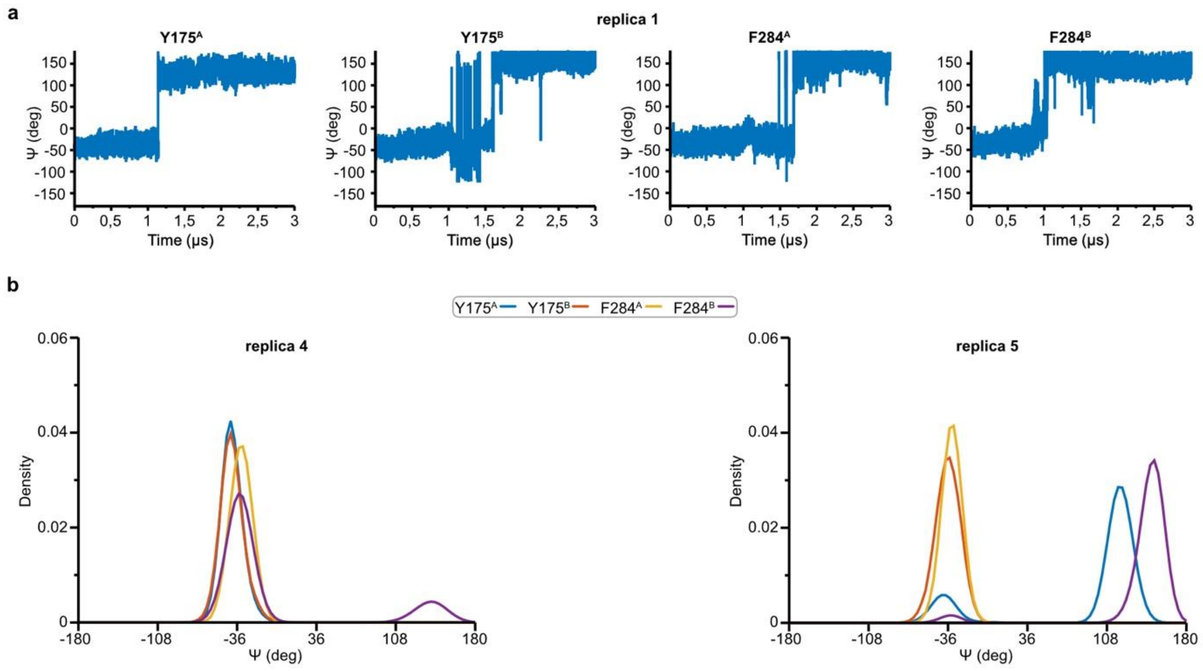

Supplementary Fig. 14. SF conformational change during the phosphorylated TREK-2* simulations. (a) Time course of psi angles (ψ) of F284 and Y175 in chain A and B during one phosphorylated TREK-2* simulation starting from the up-state (replica 1). **(b)** Distribution of psi angle (ψ) for F284 and Y175 from two phosphorylated TREK-2* simulations starting from the up-state.

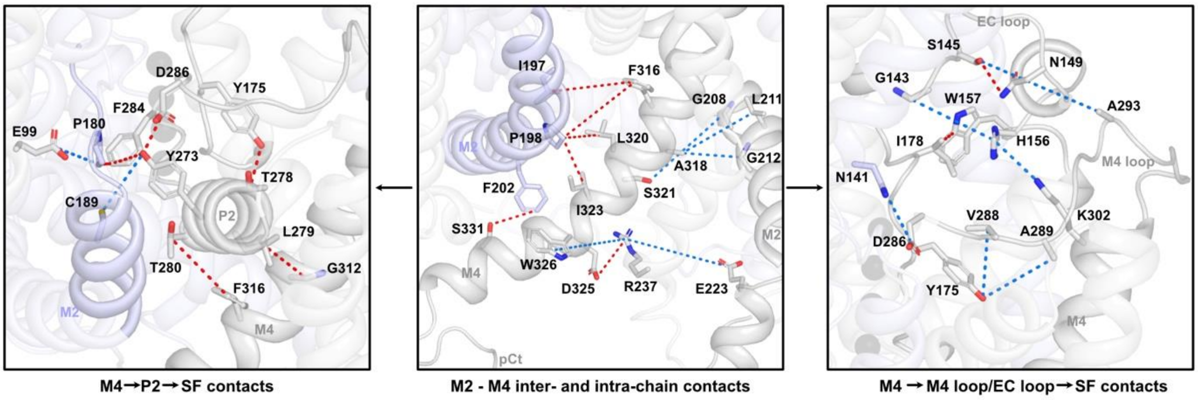

Supplementary Fig. 15. Coupling mechanism between pCt and SF. Key M2 – M4 inter- and intra-chain contacts. The first pathway of the pCt/M4 → SF coupling involves a number of M4 → P2 → SF contacts. The second pathway of the pCt/M4 → SF coupling involves the M4 → M4 loop/EC loop → SF contacts.

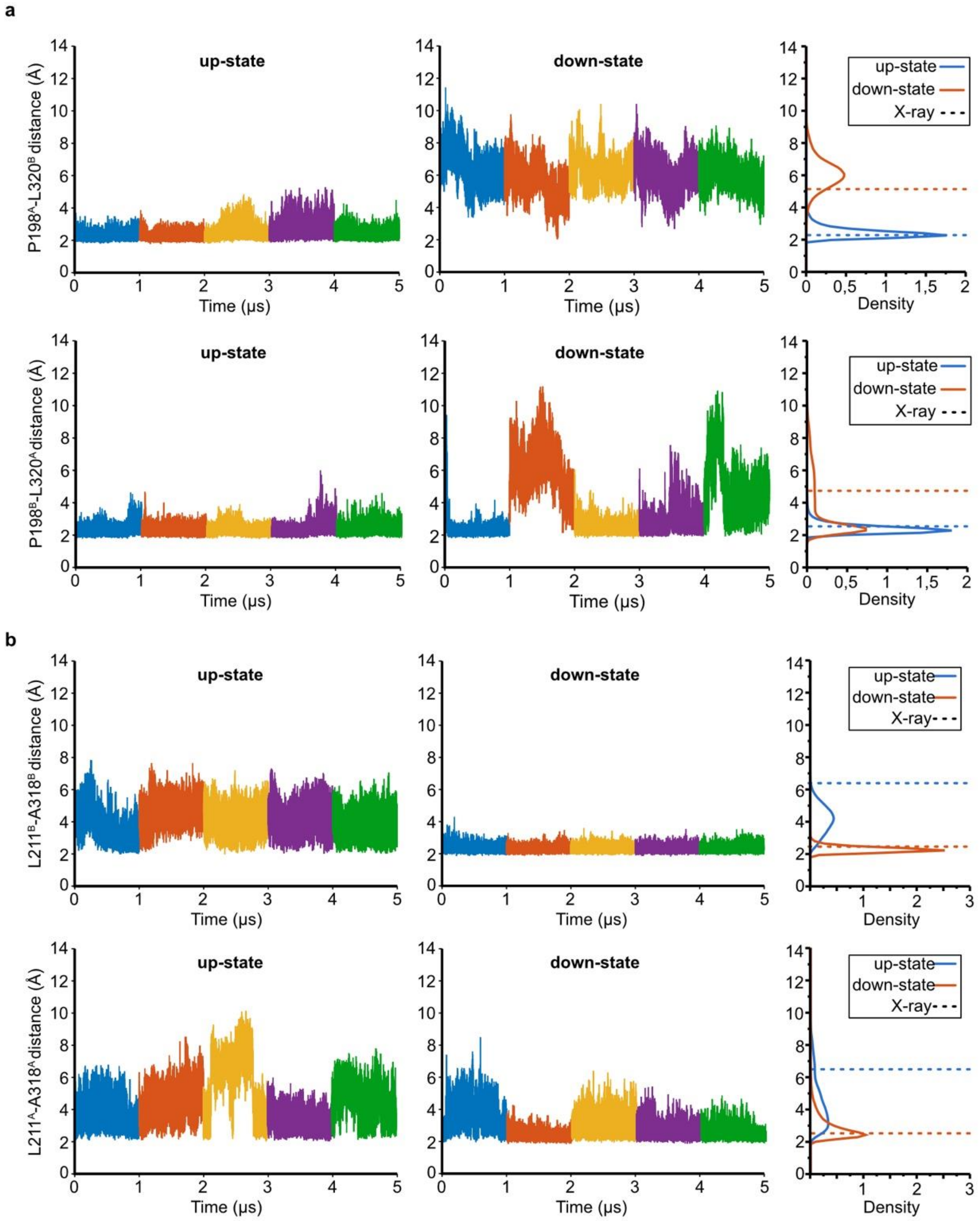

Supplementary Fig. 16. (a) Time course of the inter-chain distance between P198 and L320 and **(b)** the intra-chain distance between L211 and A318 for both up- and down-state simulations of TREK-2*. Histograms of distance distribution are included on the right site, along with the distances derived from X-ray structures shown as dashed lines.

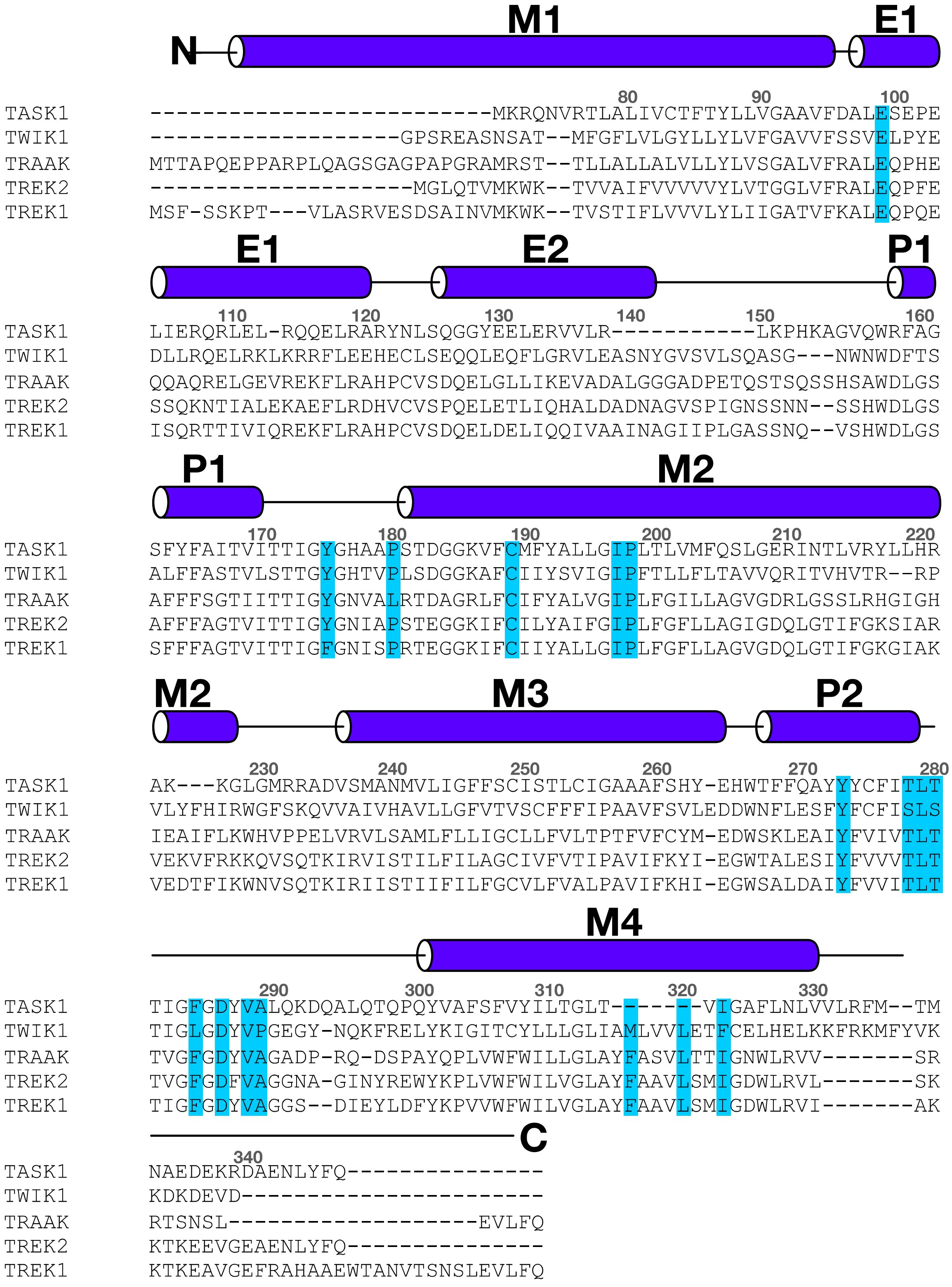

Supplementary Fig. 17. Sequence alignment of several K2P channels. Sequence alignment of TASK-1 (PDB ID: 6RV2)[4], TWIK-1 (PDB ID: 3UKM)[5], TRAAK (PDB ID: 4WFE)[6], TREK-2 (PDB ID: 4BW5)[2] and TREK-1 (PDB ID: 6CQ6)[7] was generated by CLUSTALW2[8]. Important amino acid residues identified from the interaction network analysis are highlighted in blue.

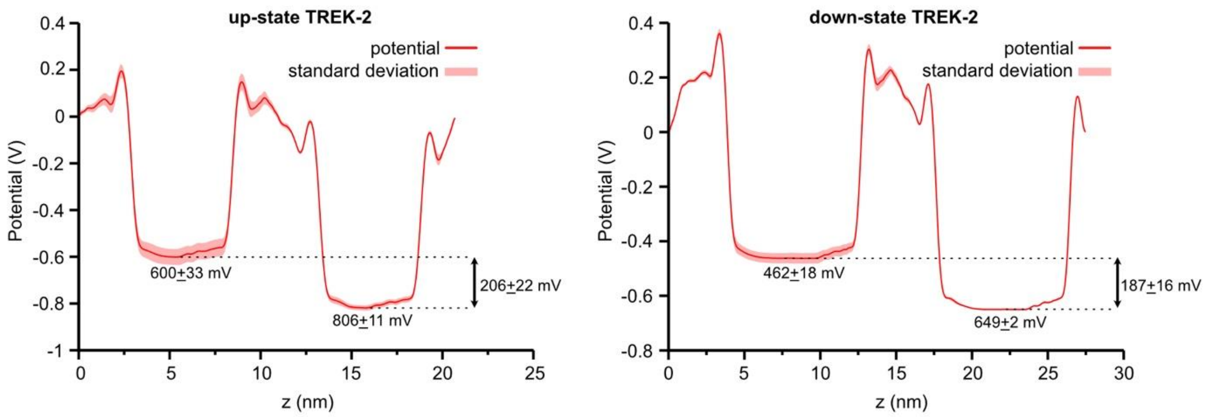

Supplementary Fig. 18. Transmembrane potentials during the MD simulations. Electrostatic potential along the z-axis for up- and down-state TREK-2* simulations. Electrostatic potentials were derived from 5 runs of 1 µs simulations with an ion imbalance of 2 between two compartments in the CompEL MD setup.

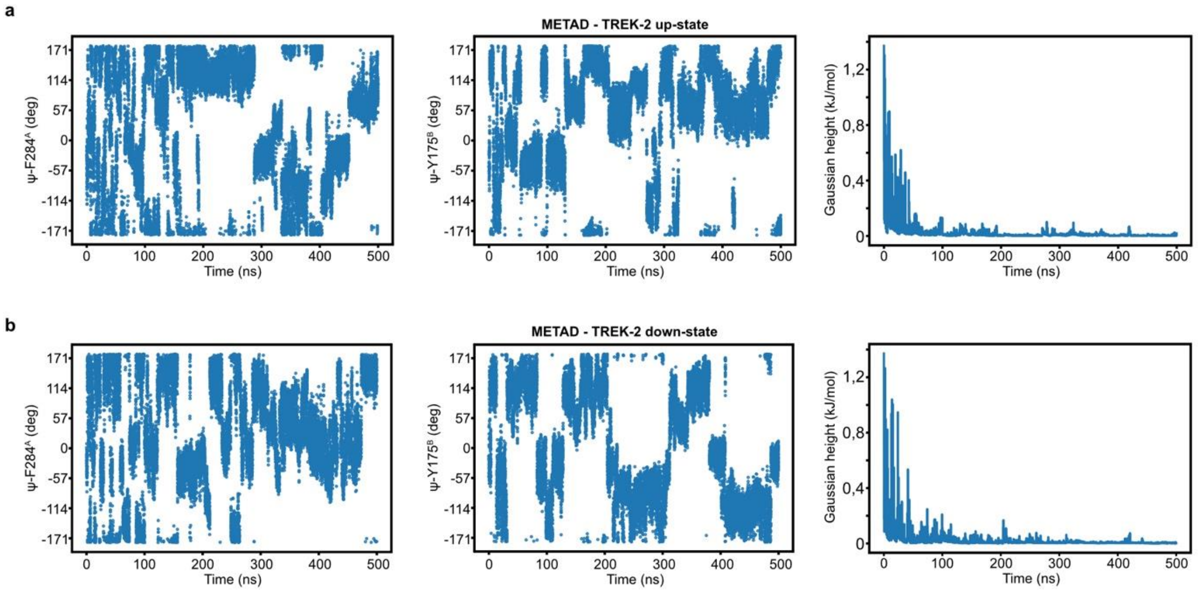

Supplementary Fig. 19. Metadynamics simulations of the up- and down-state TREK-2* channel. Psi angles (ψ) of F284^{A} and Y175^{B} over simulation time in the well-tempered metadynamics simulations of TREK-2*, starting from the **(a)** up- and **(b)** down-state, with the Gaussian potential (third coloum) added during the simulations.

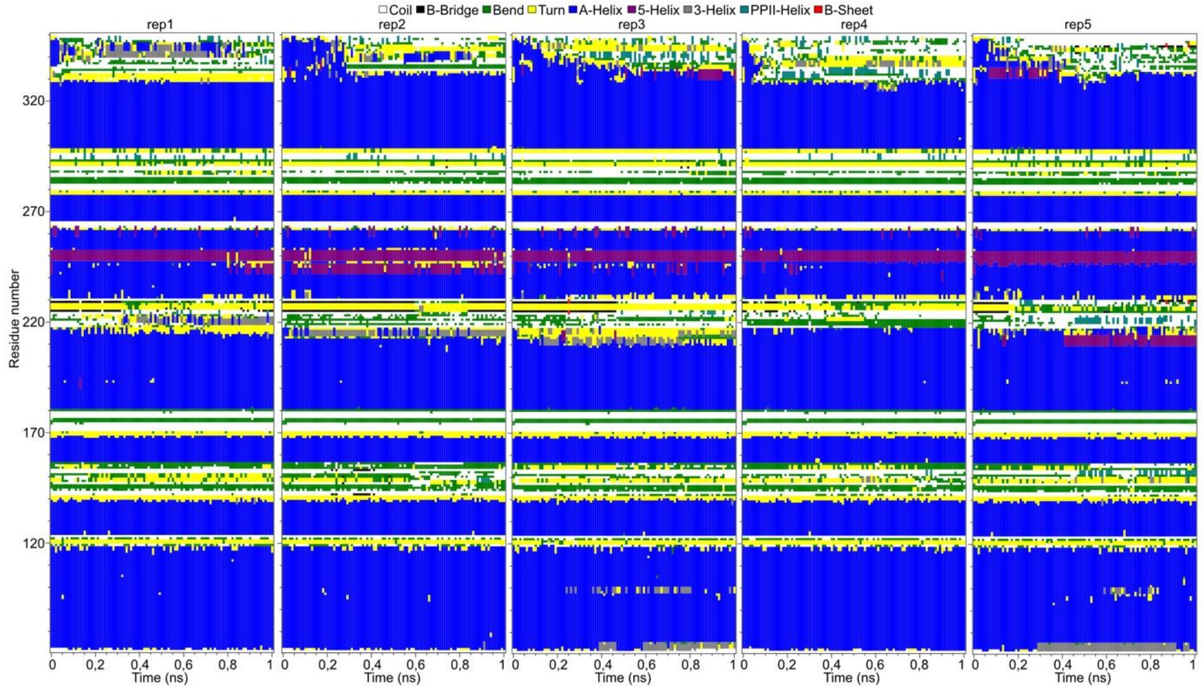

Supplementary Fig. 20. Secondary structure analysis of up-state TREK-2*. Time-dependent evolution of secondary structure calculated using gromacs DSSP tool for 5 runs of 1 µs up-state TREK-2* simulations.

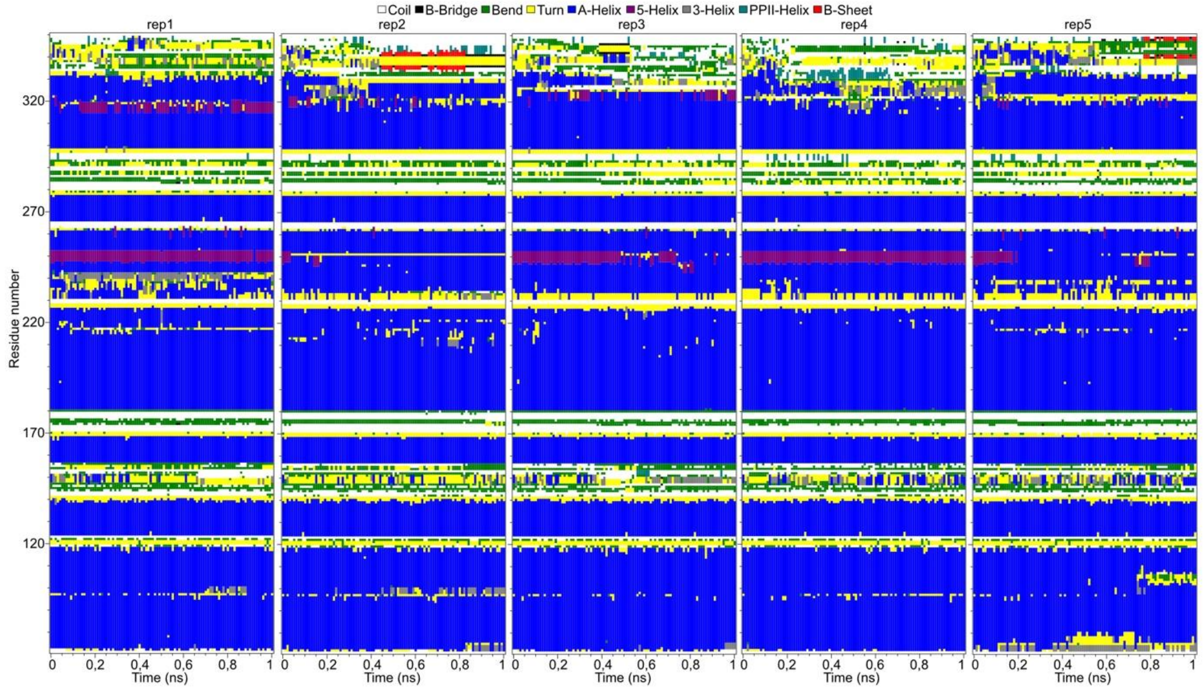

Supplementary Fig. 21. Secondary structure analysis of down-state TREK-2*. Time-dependent evolution of secondary structure calculated using gromacs DSSP tool for 5 runs of 1 μs down-state TREK-2* simulations.

Supplementary Table 1. Summary of apo TREK-2 computational electrophysiology simulations

System	State	Model	PDB ID	Δq (e^-)	Time (ns)	[C] (mM)	Vm (mV)	Ion permeations	γ (pS)
TREK-2	Up	Apo – Extended Length	4BW5	2	1000	602.2	206 $\pm$ 10	5, 6, 3, 5, 6	4 $\pm$ 0.87
TREK-2	Up	Apo – Extended Length	4BW5	2	1000	602.2	-202 $\pm$ 7	0, 1, 2, 2, 2	1.12 $\pm$ 0.6
TREK-2	Down	Apo – Extended Length	4XDJ	2	1000	598.4	192 $\pm$ 12	0, 0, 0, 0, 0	0
TREK-2	Down	Apo – Extended Length	4XDJ	2	1000	598.4	-189 $\pm$ 16	0, 0, 0, 0, 0	0

Each system contains 5 replicas. Temperature 300 K. K^+ and Cl^- ions were used. AMBER99SB-ILDN force field and SPC/E water model.

Supplementary Table 2. Contacts from the interaction network analysis. The probability was calculated as the population difference between up-state and down-state contacts. If not specified as XXX^{B}, the residues come from chain A.

Intermolecular			Intramolecular			Intramolecular		
Residue 1	Residue 2	Population difference (up – down)	Residue 1	Residue 2	Population difference (up – down)	Residue 1	Residue 2	Population difference (up – down)
S331^{B}	F202	0.698	A318	F244	0.956	G312	L279	0.323
K186^{B}	F287	0.685	D286	Y273	0.899	F252	F215	0.317
K114^{B}	S150	0.648	N149^{B}	S145^{B}	0.889	Y273	F284	0.281
A111^{B}	S150	0.612	A318^{B}	F244^{B}	0.864	F316	T280	0.259
A111^{B}	S151	0.595	Y315^{B}	I245^{B}	0.855	P303	F163	- 0.372
A114^{B}	D286	0.556	S154	N152	0.758	R237	K224	- 0.409
I110^{B}	S150	0.504	A247^{B}	F215^{B}	0.751	S181	A142	- 0.409
F316^{B}	P198	0.430	D325	R237	0.693	K302^{B}	H156^{B}	- 0.415
S155^{B}	P101	0.404	W326	T241	0.684	A289^{B}	Y175^{B}	- 0.443
F316^{B}	I197	0.399	D325^{B}	R237^{B}	0.672	C189^{B}	F165^{B}	- 0.448
I323^{B}	P198	0.339	Y315^{B}	C249^{B}	0.652	V288^{B}	Y175^{B}	- 0.452
P180^{B}	F284	0.297	I170	G167	0.627	A293^{B}	S145^{B}	- 0.454
L320^{B}	P198	0.249	F252^{B}	F215^{B}	0.627	F244	S218	- 0.490
P198^{B}	L320	0.142	F316^{B}	T280^{B}	0.608	A318	G212	- 0.517
P180^{B}	E99	- 0.247	I178^{B}	W157^{B}	0.567	H156^{B}	G143^{B}	- 0.542
C189^{B}	Y273	- 0.321	G312^{B}	L279^{B}	0.562	R227	E223	- 0.557
A111^{B}	N152	- 0.350	F244^{B}	G212^{B}	0.561	F244^{B}	S218^{B}	- 0.567
E270^{B}	K186	- 0.354	S240^{B}	E323^{B}	0.553	G312	F252	- 0.585
D118^{B}	Q106	- 0.392	Q230	R227	0.531	I236^{B}	F226^{B}	- 0.601
R328^{B}	D325	- 0.415	A247	F215	0.511	I236	V231	- 0.635
G91^{B}	Y192	- 0.447	T278^{B}	Y175^{B}	0.477	G285^{B}	G176^{B}	- 0.651
S155^{B}	K107	- 0.466	F252	L211	0.474	S151	S145	- 0.685
N141^{B}	D286	- 0.590	I239^{B}	F226^{B}	0.464	R237^{B}	E323^{B}	- 0.713
			F252^{B}	L211^{B}	0.456	I239	F226	- 0.739
			F244	G212	0.452	W326^{B}	R237^{B}	- 0.794
			K302	H156	0.442	Y297	N292	- 0.820
			Y192	I170	0.419	G196	I170	- 0.836
			A314	G208	0.414	Y297^{B}	E264^{B}	- 0.836
			F284^{B}	Y273^{B}	0.408	Y297	E264	- 0.889
			H156	G143	0.380	Y315^{B}	A247^{B}	- 0.893
			T281	T172	0.369	A318^{B}	L211^{B}	- 0.920
			G291	E264	0.360	S321^{B}	G208^{B}	- 0.936

Supplementary Table 3. Summary of unbiased MD simulations.

System	State	Model	PDB ID	Time per replica (ns)	Replica
(1) TREK-2	Up	Apo – Extended Length	4BW5	1000	5
(2) TREK-2	Down	Apo – Extended Length	4XDJ	1000	5
(3*) TREK-2	Up	Phosphorylation – Extended Length	4BW5	3000	7
(4) TREK-2	Down	Decyl-MTS – Extended Length	4XDJ	1000	5
(5) TREK-2	Down	$MTSET^+$ – Extended Length	4XDJ	1000	5
(6) TREK-2	Down	Decyl-MTS– Extended Length – Adiabatic Bias MD	4XDJ	10	5
(7) TREK-2	Down	$MTSET^+$ – Extended LengthAdiabatic Bias MD	4XDJ	10	5
(8) TREK-2	Up	Apo – Extended Length – Well Tempered Metadynamics	4BW5	500	1
(9) TREK-2	Down	Apo – Extended Length – Well Tempered Metadynamics	4XDJ	500	1

Temperature 300 K. K^+ and Cl^- ions were used. AMBER99SB-ILDN force field and SPC/E water model.
*Temperature 320K

Supplementary Table 4. Simulation details of each simulation setup.

System	*Total N of atoms*	*Total N of H_2O*	*Salt concentration*	*Total N of lipid composition (POPC – 52 atoms)*	*Box dimensions (Å)*
TREK-2 apo up-state	185508	44788	614 mM	590	103.7x103.7x207.3
TREK-2 apo down-state	244908	64440	616 mM	590	101.3x101.3x275.2
TREK-2 Decyl-MTS attached down-state	243650	64450	613 mM	598	101.6x101.6x275.2
TREK-2 MTS-ET$^+$ attached down-state	243112	64424	613 mM	590	101.4x101.4x275.5
TREK-2 phosphorylated S331 up-state	245250	64486	612 mM	594	101.5x101.5x275.3
Decyl-MTS-attached down-state – ABMD	121825	32225	613 mM	299	101.6x101.6x137.6
MTS-ET-attached down-state – ABMD	121556	32212	613 mM	295	101.4x101.4x137.7
TREK-2 apo up-state – MetaD	122626	32244	613 mM	297	101.5x101.5x137.4
TREK-2 apo down-state – MetaD	122454	32220	616 mM	295	101.3x101.3x137.6

References

1. Jumper, J. *et al.* Highly accurate protein structure prediction with AlphaFold. *Nature* **596**, 583–589 (2021).
2. Dong, Y. Y. *et al.* K2P channel gating mechanisms revealed by structures of TREK-2 and a complex with Prozac. *Science* **347**, 1256–1259 (2015).
3. Hornak, V. *et al.* Comparison of multiple Amber force fields and development of improved protein backbone parameters. *Proteins* **65**, 712–725 (2006).
4. Rödström, K. E. J. *et al.* A lower X-gate in TASK channels traps inhibitors within the vestibule. *Nature* **582**, 443–447 (2020).
5. Miller, A. N. & Long, S. B. Crystal Structure of the Human Two–Pore Domain Potassium Channel K2P1. *Science* **335**, 432–436 (2012).
6. Brohawn, S. G., Campbell, E. B. & MacKinnon, R. Physical mechanism for gating and mechanosensitivity of the human TRAAK K+ channel. *Nature* **516**, 126–130 (2014).
7. Lolicato, M. *et al.* K2P2.1 (TREK-1)–activator complexes reveal a cryptic selectivity filter binding site. *Nature* **547**, 364–368 (2017).
8. Thompson, J. D., Higgins, D. G. & Gibson, T. J. CLUSTAL W: improving the sensitivity of progressive multiple sequence alignment through sequence weighting, position-specific gap penalties and weight matrix choice.
